# The effect of the plant parts (leaf, flower, stem and seed) on antioxidant activity, bioactive compounds, fatty acids and mineral contents of chaste (*Vitex agnus-castus* L.) plant

**DOI:** 10.1007/s13197-025-06314-y

**Published:** 2025-06-04

**Authors:** Isam A. Mohamed Ahmed, Fahad Al Juhaimi, Mehmet Musa Özcan, Nurhan Uslu, Emad Karrar

**Affiliations:** 1 Department of Food Science & Nutrition, College of Food and Agricultural Sciences, King Saud University, Riyadh, Saudi Arabia; 2 Department of Food Engineering, Faculty of Agriculture, Selcuk University, 42031 Konya, Turkey; 3 Department of Plant Sciences, North Dakota State University, Fargo, 58108 ND USA

**Keywords:** Chaste, Oil, Fatty acids, Bioactive compounds, Antioxidant activity, Minerals

## Abstract

Total phenolic and flavonoid amounts of the *Vitex agnus-castus* plant were displayed to be between 1146.43 (seed) and 1724.21 mgGAE/100 g (flower) to 4250.00 (seed) and 9264.29 mg/100 g (stem), respectively. *Vitex agnus-castus* parts contained total carotenoids ranging from 3.79 (flower) to 20.81 µg/g (leaf). In addition, *Vitex agnus-castus* parts contained total carotenoids ranging from 3.79 (flower) to 20.81 µg/g (leaf). Antioxidant activities of the parts of chaste plant changed between 18.39 (seed) and 19.05 mmol/kg (stem). Statistically significant differences were detected in quantities of phenolic constituents in *Vitex agnus-castus* plants depending on the plant parts (*p* < 0.05). Catechin and rutin quantities of the parts of chaste plant were displayed to be between 185.54 (leaf) and 311.60 mg/100 g (flower) to 137.39 (flower) and 217.77 mg/100 g (stem), respectively. Oleic and linoleic acid quantities of the oils of the chaste plant parts were characterized to be between 16.62 (leaf) and 17.72% (seed) to 37.68 (leaf) and 65.21% (seed), respectively. Chaste plant parts contained the highest amounts of Ca, followed by K, S, Mg, P, Fe, Mn, Zn, B and Cu in decreasing order. A significant diversity and variability was observed between the chemical properties of Chaste plant part extracts.

**Graphical abstract:**

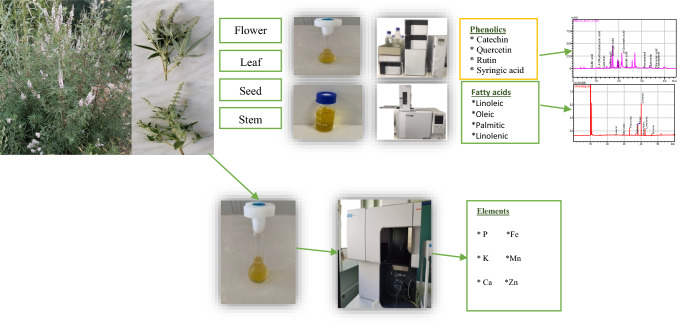

## Introduction

Chaste (*Vitex agnus-castus* L.), a shrubby plant belonging to the *Vitex* genus of the Verbenaceae family, is a deciduous plant that is distributed especially on the Mediterranean and Asian coasts and reaches a height of up to 5 m (Rani and Sharma, 2013; Ibrahim et al. 2009; Berrani et al. 2021). *V. agnus castus*, a round-topped, upright and low-branched, thin-medium textured plant, is called chaste tree, five finger herb, Karacahayit, Karahayıt, ‘Ayıd’, ‘Ayıt’, ‘Priest Pepper’ and ‘Chastity Tree’ in Turkey and has different uses (Kolancı, 2017; Özderin 2021; Kavaz et al 2022). The regions where the Chaste plant is distributed in Turkey are especially the Eastern Black Sea, Marmara, Aegean, Mediterranean and partly the Southeastern Anatolia Region (Fakir et al. 2014). The length of the plant's spike-shaped flowers, its full flower structure, its rich color range from white to light pink and purple, its continuous flowering, the formation of large seeds on the spike and its striking appearance are the features that make the plant suitable for use in landscapes (Girmen and Karagüzel, 2005).

Phenols are characterized by a hydroxyl group directly attached to an aromatic ring and are members of a group of aromatic chemical compounds with weak acidic properties (Okwu and Okwu, 2004). The fruits, flowers and leaves of *Vitex agnus-castus* are rich in bioactive substances as they contain large amounts of phenolic acids and their derivatives, flavonoids, tannins, iridoids and diterpenoids (Latovi et al. 2012; Fakir et al. 2014; Săvulescu et al. 2019; Bakr et al. 2020; Thaçi et al. 2022; Zhelev et al. 2022). Although *V. agnus castus* fruit contains various chemical constituents such as flavonoids, terpenoids and iridoids, chaste leaves are rich in polyphenols (Hoberg et al. 2001; Li et al. 2013).

In addition to being traditionally used as a lactagogue and hormone regulator, *V. agnus-castus* seeds have also been reported to have an antiaging effect on the reproductive system in vivo (Ahangarpour et al. 2016; Yalçın et al. 2021). Chaste (*Vitex* L.) leaves, bark, roots, stems, flowers, fruits and seeds have been reported to have antimicrobial effects against various microorganisms (Thaçi et al. 2022). A 5% infusion of Chaste fruits is used in Turkish folk medicine as a diuretic, carminative and sedative, while its fruits and leaves are used to protect woolen fabrics against moths (Baytop, 1999).

The leaves and fruits of the chaste plant have many biological properties such as antioxidant, spasmolytic, and it is used for rheumatic pains, sprains, as anticancer, diuretic, respiratory infections (Gökbulut et al., 2010; Zielińska and Zieliński, 2011; Rani, 2013). The goal of this study is to investigate the effect of different parts of the chaste plant (leave, flower, stem and seed) on oil, total phenolic, flavonoid, carotenoid quantities, antioxidant capacities, bioactive compounds, fatty acids and mineral contents of chaste (*Vitex agnus-castus*) plant.

## Material and methods

### Material

The parts (leaf, flower, stem and seed) of chaste (*Vitex agnus-castus* L.) were provided from Mersin-Gülnar region (Büyükeceli area) in May–September 2024. *V. agnus-castus* parts were put in paper bags, and transferred to laboratory. Plant parts were dried in airy place at room temperature.

### Methods

#### Moisture content

Moisture quantities of the chaste plant parts were assessed at 105 °C in an oven till a constant weight.

#### Carotenoid content

Extraction of the chaste plant parts for carotenoids was carried out according to the methodology proposed by Rocha et al. (2013). After pretreatments, the volume of the extracts was completed with 25 mL petroleum ether. Then, the absorbance was stated at 450 nm.

#### Extraction process

Extraction process of the chaste samples were performed according to the methodology suggested by Yushchyshena and Tsurkan (2014). After 20 ml of methanol:water (80:20, v/v) were added to 2 g powdered samples, the mixture was treated in water-bath for 30 min at 60 °C, and followed by centrifugation at 6000 rpm for 10 min. Then, the supernatant was collected and filtered.

#### Crude protein

The protein results in chaste samples was recorded by Kjeldhal method and it was determined using the following formula (AOAC 1990):

Protein content (%) = Nitrogen × 6.25.

#### Total phenolic content

Total phenolic contents of the chaste extracts were displayed by using the Folin-Ciocalteu according to the methodology applied by Yoo et al. (2004). At the end of pretreatments, the absorbace was defined at 750 nm The findings were stated as mg GAE/100 g.

#### Total flavonoid content

Total flavonoid amounts of the chaste extracts were established using colorimetric method according to the study proposed by Hogan et al. (2009). After pretreatments, the absorbance was recorded at 510 nm. The findings are depicted as mg quercetin/ 100 g.

#### Antioxidant activity

The antioxidant activity of the chaste sample was defined using DPPH (1,1-diphenyl-2-picrylhydrazyl) according to the report proposed by Lee et al. (1998). After pretreatments, the absorbance was read at 517 nm. The findings are specified as mmol TE/kg (fw).

#### Determination of phenolic compounds

HPLC (Shimadzu) equipped with PDA detector and Inertsil ODS-3 (5 µm; 4.6 × 250 mm) column was applied for analysis of phenolic constituents of the chaste plant parts. The mobile phase was a mixture of 0.05% acetic acid in water (A) and acetonitrile (B) with the flow rate of 1 ml/min at 30 °C. The injection volume was 20 µl. The peaks were taken at 280 using a PDA detector. The total running time per sample was 60 min.

#### Oil content

Each chaste plant parts were ground in the mill and turned into powder. 10 g of each sample was weighed and placed in the Soxhelt cartridge, and after closing the mouth with cotton, the cartridge was put in the extractor. Chaste samples were extracted with petroleum ether at 50 °C for 5 h, and after removing the petroleum ether in each sample with an evaporator, the oil amounts were calculated (%) (AOAC 1990).

#### Fatty acid composition

Fatty acid methyl esters of the oils of chaste parts were esterified according to the methodology applied by Multari et al. (2019), and gas chromatography (Shimadzu GC-2010, Kyoto, Japan) equipped with flame-ionization detector (FID) and capillarycolumn (Tecnocroma TR-CN100, 60 m × 0.25 mm, film thickness: 0.20 µm) was used to detect the fatty acid composition. Mobile phase was nitrogen with 1.51 ml/min flow rate. Total flow and split rates were 80 ml/min and 1/40, respectively. Column temperature was programmed 120ºC for 5 min and increased to 240 °C at 4 °C/min and held 25 min at 240 °C.

#### Element analysis of chaste tree parts

Different types of chaste tree parts, which were dried to a constant weight in a drying cabinet at 70 °C, were ground in the mill and then 0.2 g of each sample was weighed into a volumetric flask. After adding 5 ml 65% HNO_3_ and 2 ml 30% H_2_O_2_ to these weighed chaste parts, respectively, the mixture was burned at 210 °C and 200 PSI in a closed microwave system (Cem-MARS Xpress). After this process, the mixture was completed to 20 ml with distilled water and the quantitative values of the elements were characterized by the ICP-OES equipment (Tošic et al. 2015).

#### Statistical analysis

The statistical analysis of results was performed by the JMP statistical program. Statistically changes were established by the analysis of variance (ANOVA) procedure in all data *p* < 0.05).

## Results and discussion

### The chemical and bioactive properties of the flower, leaf, seed and stem parts of the chaste tree

The chemical and bioactive properties of the flower, leaf, seed and stem parts of the chaste tree collected from Gülnar (Büyükeceli) district of Mersin are defined in Table 1. The results exhibited differences based on the parts of the chaste tree. The moisture and oil quantities of the parts of *Vitex agnus castus* plant were specified to be between 5.60 (leaf) and 6.78% (stem) to 1.41 (stem) and 4.10% (seed), respectively. Total phenolic and flavonoid amounts of the *Vitex agnus-castus* plant were stated to be between 1146.43 (seed) and 1724.21 mgGAE/100 g (flower) to 4250.00 (seed) and 9264.29 mg/100 g (stem), respectively. In addition, *Vitex agnus-castus* parts contained total carotenoids ranging from 3.79 (flower) to 20.81 µg/g (leaf). Antioxidant activities of the parts of chaste plant changed between 18.39 (seed) and 19.05 mmol/kg (stem). Total carotenoid and flavonoid quantities of leaf and stem of *Vitex agnus-castus* were higher than those of flower and seed. The highest oil quantity was determined in the seed of *Vitex agnus-castus* and it was followed by flower, leaf and stem in decreasing order. The high total carotenoid and flavonoid content in the leaf and stem parts of *V. agnus-castus* plant may be due to the fact that it has a morphologically scalloped stem surface and leafs connected to this scalloped surface by a thin side branch. Total phenolic and flavonoid quantities of different parts of the chaste plant were determined to be between 4.21 (stem) and 13.11 (flower) to 0.41 (stem) and 1.84 mgGAE/100 g (flower), respectively (Berrani et al. 2021). In addition, Berrani et al. (2021) reported that antioxidant activity values of of different parts of the chaste plant changed between 0.199 to 0.612 AAE/g (Berrani et al. 2021). Antioxidant activity (DPPH assay) value of the leafs of *V. agnus-castus* L. was established as 14.25 μg/mL (Tewari et al. 2022). According to the antioxidant activity results of chaste plant with different antioxidant methods, it was revealed that the methanol extract of *V. agnus-castus* showed stronger antioxidant activity because it is rich in phenolic compounds (Zargar et al. 2011). It was monitored that the total phenol, flavonoid contents and antioxidant activities of different parts of Chaste plant were not consistent with the results of previous studies. This may be due to the growing conditions of the plant, the time when the plant parts were collected and the analytical methods used.

**Table 1:** Some chemical and bioactive properties of the parts of *Vitex agnus-castus* plant

Plant parts	Moisture content (%)			Carotenoid content (μg/g)			Oil content (%)			Total phenolic content (mg/100 g)			Total flavonoid content (mg/100 g)			Antioxidant activity (mmolTE/kg)		
Flower	6.30	±	0.15*c	3.79	±	0.07d	3.80	±	0.14	1724.21	±	20.80a	7940.48	±	105.30c	18.43	±	0.02b
Leave	5.60	±	0.05d**	20.81	±	0.02a	3.05	±	0.07	1716.27	±	9.01b	8169.05	±	57.74b	17.87	±	0.05d
Seed	6.55	±	0.08b	6.97	±	0.02c	4.10	±	0.00	1146.43	±	17.17d	4250.00	±	24.74d	18.39	±	0.04c
Stem	6.78	±	0.19a	12.25	±	0.05b	1.41	±	0.00	1611.51	±	14.55c	9264.29	±	240.32a	19.05	±	0.00a

^*^standard deviation;** values within each column followed by different letters are significantly different at *p* < 0.05

### Phenolic compounds and their amounts identified by HPLC in vitex agnus-castus plant parts

Phenolic constituents and their amounts identified by HPLC in *V. agnus-castus* plant parts are specified in Table 2. In general, it was observed that *V. agnus-castus* plant is rich in phenolic constituents. Statistically significant differences were detected in the quantities of phenolic constituents in *V. agnus-castus* plants depending on the plant parts (*p* < 0.05). Catechin and rutin quantities of the parts of *Vitex agnus-castus* plant were displayed to be between 185.54 (leaf) and 311.60 mg/100 g (flower) to 137.39 (flower) and 217.77 mg/100 g (stem), respectively. *p*-coumaric acid and quercetin quantities of chaste (*V. agnus-castus*) parts were depicted to be between 32.71 (seed) and 151.06 mg/100 g (leaf) to 43.39 (seed) and 301.13 mg/100 g (leaf), respectively. While 3,4-dihydroxybenzoic acid amounts of the chaste plant parts are defined between 50.11 (seed) and 89.27 mg/100 g (stem), syringic acid contents of the parts of the chaste plant were assigned to be between 35.42 (seed) and 138.88 mg/100 g (flower). The highest resveratrol (38.82) and ferulic acid (366.42 mg/100 g) were established at the stem part of *Vitex agnus-castus* plant. Caffeic acid amounts of *Vitex agnus-castus* plant were defined to be between 30.79 (seed) and 58.39 mg/100 g (flower). The lowest amount of phenolic compound in the parts of the chaste plant was cinnamic acid. In general, the plant part with the highest phenolic compound was the leaf, followed by the stem, flower and seed parts in decreasing order. The mixture of *V. agnus-castus* plant contained 1603.88 mg/kg 4-hydroxybenzoic acid, 58.63 mg/kg 3-4-Dihydroxy benzaldehyde, 668.49 gentisic acid, 4906. 02 protocatechuic acid, 69.56 vanillic acid, 84.29 caffeic acid, 8.97 ferulic acid (Özderin 2021). Different parts (leaf, root, stem, flower and seed) of the plant *Vitex agnus-castus* contained 20.20 (root) to 12,077*.*1 μg/kg (leaf) gallic acid, 0.68 (leave) to 1.44 (seed) catechin, 0.01 (root) to 0.26 (leaf and flower) rutin, 42,019*.*9 (leave) to 295,098 (root) chlorogenic acid, 44,277.2 (seed) to 27,230.3 (leave) caffeic acid, 2.38 (root) to 450.97 (flower) quercetin, 9.28 (flower) to 78.17 (leaf) syringic acid, 10,937(root) to 22,771 (seed) p-hydroxybenzoic acid, 2.95 (root) to 525.96 μg/kg (stem) ferulic acid (Berrani et al. 2021). Demirtaş and Pişkin (2020) determined the amounts of gallic acid, caffeic acid, lueolin and *p*-coumaric acid as 126.9, 63.3, 344.1 and 15.6 μg/g in *V agnus-castus* L fruit extract. In previous study, Parlak et al (2016) identified 0.281 μg/g gallic acid, 21.506 μg/g 4-Hydroxy benzoic acid, 0.647 μg/g caffeic acid and 0.122 μg/g ferulic acid in *V. agnus-castus* seed samples collected from Denizli location in Turkey. In another study, Ceviz (2016) detected 0.02 gallic acid, 0.06 protocatechuic acid, 0.95 p-hydroxybenzoic acid, 0.57 chlorogenic acid, 0.02 epicatechin, 0.02 syringic acid, 0.01 vanillin, 0.04 *p*-coumaric acid, 0.21 benzoic acid, 0.01 cinnamic acid, 0.02 quercetin, 0.04 luteolin and 0.10 mg/g kaempferol in the mixture of branch and stem samples of *V. agnuscastus* (Chaste). Kawashty et al. (2016) pointed out that *V. trifolia* L. sample contained 1.92 mg/100 g gallic acid, 2.21 protocatechuic acid, 4.68 chlorogenic acid, 1.58 caffeic acid, 8.56 vanillic acid, 2.43 *p*-coumaric acid, 6.00 ferulic acid, 2.12 iso-ferulic acid, 9.29 ellagic acid, 9.31 salicylic acid, 1.24 o-coumaric acid, 35.41 vanillic acid and 16.38 mg 100 g (dw) 4-hydroxy benzoic acid. Our findings regarding phenolic component amountsof the chaste parts were defined different than those defined by Kawashty et al. (2016) in *V. trifolia* L. sample, Demirtaş and Pişkin (2020), Özderin (2021) and Berrani et al. (2021). It has been explained that the main factors affecting the amounts of phenolic compounds in plants are growing conditions, processing factors, genetic, ripening process, harmful air pollution, extreme temperatures and stress conditions (Figueiredo et al. 2008).

**Table 2:** Phenolic compounds of the parts of *Vitex agnus-castus* plant

Phenolic compounds (mg/100 g)	Flower			Leave			Seed			Stem		
Gallic acid	61.96	±	3.29*c	68.03	±	2.48b	50.11	±	1.17d	89.27	±	4.84a
3,4-Dihydroxybenzoic acid	130.68	±	6.65b**	139.50	±	10.65a	75.13	±	2.40c	12.03	±	0.17d
Catechin	311.60	±	22.44d	185.54	±	12.11c	188.18	±	11.89b	283.94	±	23.17a
Caffeic acid	58.39	±	4.01a	42.48	±	1.12b	30.79	±	2.16d	39.66	±	0.54c
Syringic acid	138.88	±	10.61a	69.63	±	3.71b	35.42	±	3.10d	53.03	±	0.44c
Rutin	137.39	±	9.21d	186.28	±	13.59bc	174.22	±	15.99	217.77	±	13.06a
p-Coumaric acid	40.73	±	0.32c	151.06	±	16.46a	32.71	±	0.20d	149.30	±	10.80b
Ferulic acid	18.35	±	0.34c	32.16	±	0.12b	12.90	±	0.93d	366.42	±	28.55a
Resveratrol	17.17	±	1.15c	34.20	±	0.72b	14.85	±	0.99d	38.82	±	3.61a
Quercetin	189.53	±	10.56b	301.13	±	21.23a	43.39	±	1.03d	53.48	±	3.69c
Cinnamic acid	5.83	±	0.16b	8.78	±	0.27a	3.77	±	0.07c	2.30	±	0.22d
Kaempferol	35.48	±	1.02a	25.38	±	0.46b	4.46	±	0.22d	10.30	±	1.63c

^*^standard deviation;** values within each row followed by different letters are significantly different at *p* < 0.05

### Fatty acids and amounts of the oils of chaste tree parts

Fatty acids and amounts of thechaste tree parts are depicted in Table 3. The fatty acids that showed the most fluctuations depending on the chaste tree parts were palmitic, linoleic, arachidic, linolenic and erucic acid. It is important for the consumer that the erucic acid content of seed and stem oils within the chaste tree parts is below 5% and is found in very low amounts. Palmitic, arachidic, linolenic and erucic acid quantities of seed and stem parts were found in much lower amounts than those of flower and leaf parts, while oleic and linoleic acid contents were assessed in much higher levels. The dominant fatty acids of the oils of the chaste tree parts were linoleic, oleic, palmitic, linolenic and stearic acids. Oleic and linoleic acid quantities of the oils of the chaste plant parts were characterized to be between 16.62 (leaf) and 17.72% (seed) to 37.68 (leaf) and 65.21% (seed), respectively. Also, palmitic and stearic acid quantities of the oils of the chaste parts were defined to be between 6.46 (seed) and 13.93% (flower) to 2.55 (stem) and 4.63% (seed), respectively. Compared to most other oilseeds, the linolenic acid content of the oils from the chaste tree parts was found to be quite high and the linolenic acid contents of the flower, leaf, seed and stem parts of the chaste tree were determined as 11.86%, 15.06, 3.34 and 11.72%, respectively. The stearic acid contents of the oils from the chaste tree parts varied between 2.55% (stem) and 4.63% (seed). The oil of chaste tree fruit contained 0.43 lauric, 2.64 myristic, 21.01 palmitic, 26.11 oleic, 24.76 linoleic, 5.92 linolenic, 1.53 arachidic and 2.20% behenic acids (Ibrahim et al. 2009). GLC of fatty acid methyl esters revealed that oleic (16.62 (leaf) and 17.72% (seed) and linoleic acid (37.68 (leaf) and 65.21% (seed)) were the major unsaturated fatty acids, while palmitic acid (6.46 (seed) and 13.93%) was the major saturated fatty acid. Our results with the fatty acid composition of chaste seed oil were found to be lower than the fatty acids (lauric, myristic, palmitic, oleic, arachidic, linolenic and behenic acids) of chaste fruits performed by Ibrahim et al (2009), while the amount of linoleic acid was found to be approximately 38% higher than the linoleic acid content of Ibrahim et al. (2009).

**Table 3:** Fatty acid composition of the oils extracted from of the parts of Vitex agnus-castus plant

Fatty acids (%)	Flower			Leave			Seed			Stem		
Lauric	0.56	±	0.19b	2.95	±	0.31a	0.25	±	0.01d	0.53	±	0.03c
Myristic	0.50	±	0.03c	1.86	±	0.18a	0.49	±	0.02 cd	0.56	±	0.04b
Palmitic	13.93	±	0.62a	11.19	±	0.42b	6.46	±	0.07d	10.75	±	0.05c
Stearic	3.25	±	0.03b	2.77	±	0.03c	4.63	±	0.00a	2.55	±	0.00 cd
Oleic	22.29	±	0.17a	16.62	±	0.22d	17.72	±	0.11b	17.32	±	0.07c
Linoleic	37.85	±	0.59c	37.68	±	0.57 cd	65.21	±	0.16a	53.21	±	0.46b
Arachidic	3.63	±	2.20a	3.60	±	1.25ab	0.77	±	0.53c	0.65	±	0.56d
Linolenic	11.86	±	0.09b	15.06	±	0.11a	3.34	±	0.01d	11.72	±	0.14c
Behenic	ND***			ND			0.17	±	0.01	ND		
Erucic	6.13	±	0.66b	8.26	±	0.58a	0.96	±	0.16d	2.72	±	0.09c

^*^standard deviation;** values within each row followed by different letters are significantly different at P < 0.05;***ND:nondetected

## Calculation of lipid ındices of chaste seed oil

In this study, the plant parts affected the lipid index values of the chaste part structure (Table 4). In addition, Nutritive Value Index (NVI) and Atherogenic Index results of the oils obtained from *V. agnus-castus* plant parts were specified to be between 1.73 (leaf) and 3.46 (seed) to 0.49 (seed) and 1.30 (leaf), respectively. Also, thrombogenic Index values of date seed oils were characterized to be between 0.20 (stem) and 0.27 (flower). The plant parts affected the percentage of saturated and unsaturated fatty acids. In general, plant parts had an effect on NVI results for all samples. The thrombogenic index values of chaste leaf and fruit parts were found to be similar, and the TI values of other chaste parts (stem and flower) were found to be close to those of the leave and fruit parts. Ʃ PUFA/ Ʃ SFA is an index normally used to assess the impact of diet on cardiovascular health (Chen and Liu, 2020). Ʃ PUFA/ Ʃ SFA ratio is below 0.45 Foods that are food have been considered undesirable for human nutrition (Gluchowski et al. 2020). The significant diversity in index values depending on the fatty acid composition of the research material may be due to the genetic structure of the material, growing conditions and plant parts.

**Table 4:** Fatty acid Indices for theoils extracted from the parts of Vitex agnus-castus plant

Lipid indices	Flower	Leave	Fruit (seed)	Stem
Nutritive value Index	1.83	1.73	3.46	1.85
Atherogenic Index	0.74	1.30	0.49	0.78
Thrombogenic Index	0.27	0.22	0.22	0.20

### The protein and mineral amounts of the flower, leaf, seed and stem parts of the Vitex agnus-castus plant

The protein contents of the flower, leaf, seed and stem parts of the chaste plant and the macro and microelement quantities determined by ICP-OES are specified in Table 5. The protein, macro and microelement amounts of the *V. agnus-castus* plant differed depending on the plant parts. Chaste plant parts contained the highest amounts of Ca, followed by K, S, Mg, P, Fe, Mn, Zn, B and Cu in decreasing order. The protein amounts of the parts of the chaste plant were assigned to be between 6.92 (seed) and 15.87% (flower). The highest amount of protein was found in the leaf part of the plant, followed by the flower, stem and seed in decreasing order. Calcium and potassium amounts of the parts of *Vitex agnus-castus* plant were defined to be between 8815.69 (seed) and 12,633.03 mg/kg (leaf) to 5880.26 (stem) and 10,589.19 mg/kg (flower), respectively. In addition, the microelements found in the highest amounts in the plant parts were Fe and Mn, and the Fe and Mn contents of *Vitex agnus-castus* parts were found between 92.96 (stem) and 364.77 mg/kg (leaf) to 24.07 (seed) and 32.47 mg/kg (leaf), respectively. Zn amounts of the parts of the chaste plant changed between 13.91 (stem) and 28.36 mg/kg (flower). In general, the lowest macro and microelement amounts in *Vitex agnus-castus* plant were determined in the stem part (except Ca and Mn). The differences in protein and element contents of different parts of the chaste plant may be due to the nutrient element content of the soil in which it grows, the moisture status of the soil, the physiological properties of the plant nutrient transport organs and the distribution rates of the nutrient elements to the plant parts. Crude protein content of chaste fruit was determined as 6.65% (Ibrahim et al. 2009). The protein content of our samples was higher than the result of Ibrahim et al (2009), but that of the fruit was found to be partially similar.

**Table 5:** The protein contents (%) and mineral (mg/kg) profiles and their amounts of *Vitex agnus-castus* plant parts

Plant parts	Protein	P	K	Ca	Mg	S	Fe	Cu	Mn	B	Zn
Flower	14.30 ± 1.40b*	2966.46 ± 100.74a	10,589.19 ± 305.41a	9792.66 ± 302.02c	2871.31 ± 56.69a	7642.07 ± 172.53b	147.55 ± 0.46c	6.15 ± 0.32a	26.91 ± 0.22b	24.06 ± 0.80b	28.36 ± 1.17a
Leave	15.87 ± 1.31*a	1465.33 ± 7.45c	6982.92 ± 40.27c	12,633.03 ± 171.35a	2210.94 ± 19.16b	8281.88 ± 21.86a	364.77 ± 5.51a	5.94 ± 0.27b	32.47 ± 0.35a	10.09 ± 0.32d	14.18 ± 0.03c
Seed	6.92 ± 0.28d	2027.73 ± 5.72b	8048.89 ± 57.13b	8815.69 ± 83.39d	2056.34 ± 5.60c	5363.38 ± 68.96c	170.73 ± 0.12b	5.67 ± 0.02bc	24.07 ± 0.49d	25.49 ± 0.96a	22.17 ± 0.49b
Stem	9.75 ± 003c	592.04 ± 20.75d	5880.26 ± 483.86d	10,226.59 ± 60.16b	1168.88 ± 25.43d	3840.34 ± 92.56d	92.96 ± 2.47d	4.15 ± 0.16c	25.80 ± 0.07c	13.48 ± 0.51c	13.91 ± 0.02d

^*^standard deviation;** values within each column followed by different letters are significantly different at *p* < 0.05

### The principal component analysis (PCA)

**Fig. 1:**
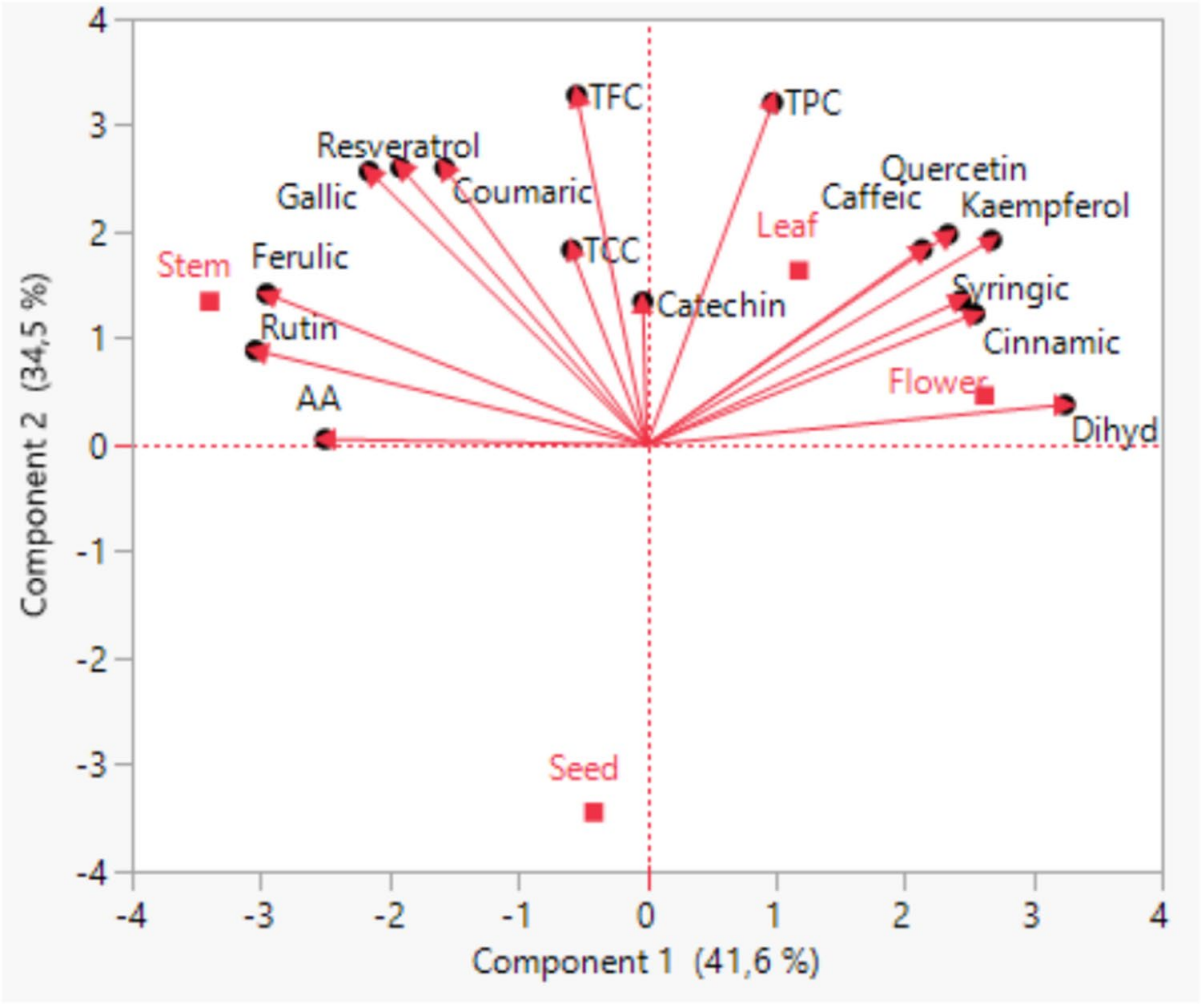
Biplot graph drawn with results of PCA (TCC: Total carotenoid content, TPC: Total phenolic content, TFC: Total flavonoid content, AA: Antioxidant activity)

The principal component analysis (PCA) of different parts of *V. agnus-castus* in relation to phenolic compounds, total carotenoid, total phenolic, total flavonoid amounts and antioxidant activity are shown in Fig. 1. The first two PCs explain 41.630% and 34.520% of total variance, respectively. A high positive correlation with PC1 was observed in 3,4-dihydroxybenzoic acid (0.957), while rutin (-0.895) and ferulic acid (-0.869) had negative correlation with PC1. Moreover, PC2 was identified with total phenolic (0.946) and total flavonoid quantities (0.966). Leaf and flower of *V. agnus- castus* was located in the positive area of both PC1 and PC2, and also contained higher quantities of phenolics placed in the same area. In addition, the stem was located in negative area of PC1 and in the positive area of PC2, and also the highest contents of rutin and ferulic acid were determined.

## Conclusion

The highest oil content was determined in the seed of *Vitex agnus-castus* and it was followed by flower, leaf and stem in decreasing order. Total carotenoid and flavonoid amounts of leaf and stem of *Vitex agnus-castus* were higher than those of flower and seed. In general, it was observed that *Vitex agnus-castus* plant is rich in phenolic compounds. The lowest amount of phenolic compound in the parts of the chaste plant was cinnamic acid. In general, the plant part with the highest phenolic compound was the leaf, followed by the stem, flower and seed parts in decreasing order. It is important for the consumer that the erucic acid content of seed and stem oils within the chaste tree parts is below 5% and is found in very low amounts. Palmitic, arachidic, linolenic and erucic acid quantities of seed and stem parts were found in much lower amounts than those of flower and leaf parts, while oleic and linoleic acid contents were found in much higher levels. Chaste plant parts contained the highest amounts of Ca, followed by K, S, Mg, P, Fe, Mn, Zn, B and Cu in decreasing order. In general, the lowest macro and microelement quantities in *Vitex agnus-castus* plant were determined in the stem part (except Ca and Mn). A significant diversity and variability was observed between the chemical properties of Chaste plant part extracts. 

## Data Availability

The data have been reviewed depending on the literature.

## Code availability

There is no special software and code application.
